# Parental SIRT1 Overexpression Attenuate Metabolic Disorders Due to Maternal High-Fat Feeding

**DOI:** 10.3390/ijms21197342

**Published:** 2020-10-05

**Authors:** Long T. Nguyen, Sonia Saad, Hui Chen, Carol A. Pollock

**Affiliations:** 1 Renal medicine, Kolling Institute, Royal North Shore Hospital, University of Sydney, Sydney, NSW 2065, Australia; carol.pollock@sydney.edu.au; 2 School of Life Sciences, Faculty of Science, University of Technology Sydney, Sydney, NSW 2007, Australia; sonia.saad@sydney.edu.au (S.S.); hui.chen-1@uts.edu.au (H.C.)

**Keywords:** obesity, sirtuin, developmental programming, metabolism, inflammation

## Abstract

Maternal obesity can contribute to the development of obesity and related metabolic disorders in progeny. Sirtuin (SIRT)1, an essential regulator of metabolism and stress responses, has recently emerged as an important modifying factor of developmental programming. In this study, to elucidate the effects of parental SIRT1 overexpression on offspring mechanism, four experimental groups were included: (1) Chow-fed wild-type (WT)-dam × Chow-fed WT-sire; (2) High-fat diet (HFD)-fed WT-dam × Chow-fed WT-sire; (3) HFD-fed hemizygous SIRT1-transgenic (Tg)-dam × Chow-fed WT-sire; and (4) HFD-fed WT dam × Chow-fed Tg-sire. Our results indicate that Tg breeders had lower body weight and fat mass compared to WT counterparts and gave birth to WT offspring with reductions in body weight, adiposity and hyperlipidaemia compared to those born of WT parents. Maternal SIRT1 overexpression also reversed glucose intolerance, and normalised abnormal fat morphology and the expression of dysregulated lipid metabolism markers, including SIRT1. Despite having persistent hepatic steatosis, offspring born to Tg parents showed an improved balance of hepatic glucose/lipid metabolic markers, as well as reduced levels of inflammatory markers and TGF-β/Smad3 fibrotic signalling. Collectively, the data suggest that parental SIRT1 overexpression can ameliorate adverse metabolic programming effects by maternal obesity.

## 1. Introduction

Obesity is a global health concern, affecting more than 650 million people worldwide and considered as one of the major risk factors of type 2 diabetes (T2D), hypertension and other metabolism-related chronic diseases [1,2]. Not only has the incidence of obesity in adults tripled in the last four decades, but also the increased number of children and adolescents with obesity is of concern. Apart from the unarguable roles of socioeconomic and genetic factors that predispose individuals of certain races and countries to the development of obesity, an emerging hypothesis for the pandemic of obesity and related diseases is the process of developmental programming, which is defined as a multigenerational transmission of diseases due to adverse environmental stimuli prior to and during pregnancy. Women of reproductive age with obesity and metabolic derangements are more likely to give birth to larger infants, who are predisposed to the development of obesity and comorbidities in later life. Interestingly, although disease transmission via maternal linage has been demonstrated to be the predominant pathway in foetal programming, an increasing number of reports also indicated the potent contributions of paternal factors, such as paternal obesity or stress, to the overall formulation of the offspring’s phenotype [3]. While a clear understanding of such interactive, multigenerational processes has not yet been achieved, conventional clinical approaches to limit their effects, such as via nutritional intervention and/or management of gestational weight gain, have been largely unsuccessful. As such, it is imperative to explore alternative treatment approaches to limit the adverse transgenerational impact of maternal obesity.

A number of recent studies, including our own, have revealed the role of sirtuin (SIRT)1, an important metabolic sensor and epigenetic regulator, in maternal obesity-induced developmental programming [4]. SIRT1 is the first member of the sirtuin family, consisting of seven members (SIRT1-7) with the primary action of deacetylating histones and non-histone proteins [5]. The full function of SIRT1 is not clearly understood, but the bulk of evidence suggests its important roles in metabolic regulation and stress responses, particularly with regards to caloric restriction and anti-aging [6,7,8]. Generally speaking, SIRT1 is upregulated by nutritional deprivation and downregulated by nutritional excess. With relevance to foetal programming by maternal obesity, our previous studies also indicated that SIRT1 expression and activity were downregulated in the offspring born to high fat diet (HFD)-fed dams, the reversal of which via SIRT1 overexpression or activation was shown to directly benefit their metabolic outcomes and reduce the risk of associated disorders later in life [9,10]. Meanwhile, other studies also demonstrated reduced SIRT1 expression in the placenta and oocytes of obese mothers, which is linked to defective oocytes, placental stress and suboptimal intrauterine nutrition [11,12,13,14,15]. Intriguingly, zygotic SIRT1 expression derived from oocytes can be modulated by paternal factors such as microRNAs in sperm [3]. As such, interventions in either mothers or fathers may have favourable impacts on foetal SIRT1 regulation and the associated metabolic pathways, hence reducing the risk of developing obesity and metabolic disorders such as type 2 diabetes and non-alcoholic fatty liver disease. The aim of this study is to examine whether maternal or paternal SIRT1 overexpression can ameliorate maternal obesity-induced metabolic consequences in the offspring.

## 2. Results

### 2.1. SIRT1 Overexpression Reduced Body Weight and Visceral Fat Mass in Dams and Sires

**Figure 1:**
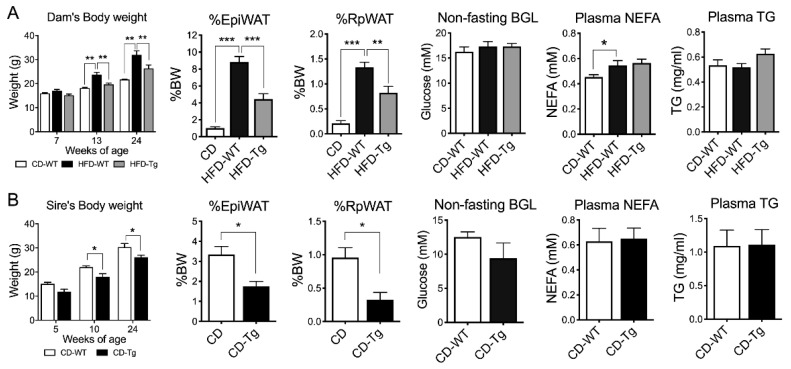
Body weight and fat mass of F0. (**A**) Dams (*n* = 6–7). (**B**) sires (*n* = 3–5). WT: Wild-type, Tg: Transgenic, EpiWAT: Epididymal white adipose tissue), RpWAT: Retroperitoneal white adipose tissue), BGL: blood glucose level, NEFA: non-esterified fatty acid, TG: triglyceride. * *p* < 0.05, ** *p* < 0.01, *** *p* < 0.001.

HFD-fed female mice showed consistently higher body weights (BW) before mating (6 weeks on HFD) and after lactation (week 24) (*p* < 0.01, Figure 1A). Fat mass and plasma non-esterified fatty acid (NEFA) levels were also significantly increased (*p* < 0.001 and *p* < 0.05 respectively) in HFD-fed dams compared to Chow-fed dams, while blood glucose level (BGL) and triglyceride levels were unchanged. SIRT1 overexpression (Tg) in HFD-fed dams significantly suppressed body weight and fat mass (*p* < 0.001, Figure 1A), but had no effects on NEFA and triglyceride levels. Similarly, SIRT1-Tg sires had lower body weight and fat mass at week 24 (*p* < 0.05, Figure 1B), but similar BGL, NEFA and triglyceride levels compared to their wild-type (WT) counterparts (Figure 1B).

### 2.2. The Effects of Parental SIRT1 Overexpression on Anthropometric and Metabolic Profiles in Offspring

Consistent with our previous findings, WT male offspring born to HFD-fed dams (MHF) had higher body weights (BW) compared to those born to Chow-fed dams (MC, *p* < 0.001, Table 1). Both epididymal (Epi) and retroperitoneal (Rp) white adipose tissue (WAT) mass (g or %BW) were significantly increased by maternal HFD consumption (*p* < 0.001). Similarly, liver weight (g or %BW) was also higher in the MHF offspring (*p* < 0.001 and *p* < 0.01, respectively). The MHF offspring’s body weight was significantly reduced if they had either a Tg dam (MTg, *p* < 0.001) or sire (PTg, *p* < 0.001). Epi WAT mass was also significantly reduced by maternal or paternal SIRT1 overexpression (*p* < 0.01). Rp WAT mass, on the other hand, was only suppressed when the dam was transgenic (*p* < 0.05). Both interventions reduced liver net weight, but only paternal SIRT1 overexpression suppressed liver weight to body weight ratio (*p* < 0.01).

Estimated milk intake was higher (*p* < 0.01, Table 1) in the offspring as a result of maternal HFD consumption, which was reversed in those born to Tg dams (*p* < 0.01). The increase in milk consumption in MHF offspring was coupled with higher levels of leptin in the circulation and a significant upregulation of hypothalamic anorexigenic neuropeptide Proopiomelanocortin (Pomc) when fasted (*p* < 0.05, Supplementary Figure S1). Maternal SIRT1 overexpression significantly normalised the level of milk intake and normalised the mRNA expressions of both orexigenic neuropeptide Y (Npy) and Pomc in MHF offspring (*p* < 0.05 and *p* < 0.05). In comparison, paternal SIRT1 overexpression had no effect on the regulation of Npy, but significantly improved the levels of Sirt1 and leptin receptor (Rb-Ob) in the offspring’s hypothalamus (*p* < 0.05).

Fasting BGL and glucose intolerance were also higher as a result of maternal HFD consumption (*p* < 0.001). Both fasting insulin levels and insulin resistance index (HOMA-IR) were increased in MHF offspring (*p* < 0.05 and *p* < 0.01 respectively). In addition, the circulating levels of triglyceride and NEFA were significantly higher compared to the control group (*p* < 0.01 and *p* < 0.001 respectively). While having no effects on the MHF offspring’s BGL, insulin level and resistance index, maternal (but not paternal) SIRT1 overexpression significantly attenuated glucose intolerance (*p* < 0.05). Both interventions significantly decreased triglyceride (*p* < 0.05) and NEFA levels (*p* < 0.001) in MHF offspring’s blood.

### 2.3. Maternal SIRT1 Overexpression Improved Adipocyte Morphology in MHF Offspring

**Figure 2:**
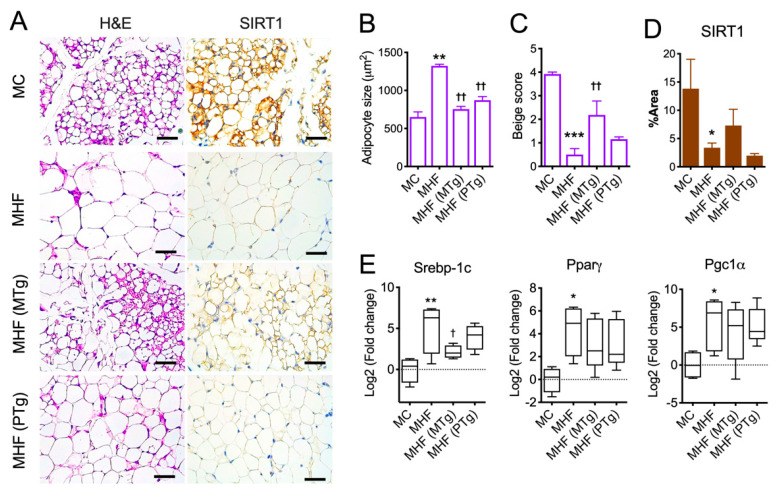
Parental SIRT1 overexpression attenuates adipocyte hypertrophy and lipid dysregulation in epidydimal fat in MHF offspring. (**A**) H&E staining (purple) and SIRT1 immunohistochemistry (brown) of Epi fat. Scale bar = 200 μm. (**B**) Adipocyte size. (**C**) Adipocyte beige score. (**D**) SIRT1 expression. (**E**) mRNA expression of metabolic regulators. *n* = 3–4. vs. MC: * *p* < 0.05, ** *p* < 0.01, *** *p* < 0.001; vs. MHF: † *p* < 0.05, †† *p* < 0.01.

Consistent with the increased WAT mass, maternal HFD consumption accelerated adipocyte growth (*p* < 0.01) and the formation of uniocular adipocytes (*p* < 0.001, Figure 2A–C), which have been associated with lower metabolic rates and insulin resistance. Both maternal and paternal SIRT1 overexpression significantly suppressed adipocyte overgrowth; however, only the former significantly improved the number of multilocular adipocytes (*p* < 0.05), a process also known as beiging.

In association with impaired adipocyte morphology, Epi WAT in MHF offspring manifested a significant reduction in SIRT1 protein expression (Figure 2A,D), coupled with significant elevations in mRNA expression of Sterol regulatory element-binding protein 1 (*SREBP1c*, *p* < 0.01), a lipogenic marker. There was also upregulation in the mRNA expression of Peroxisome proliferator-activated receptor-γ (*PPARγ*, *p* < 0.05), an essential regulator of adipocyte differentiation, and Peroxisome proliferator-activated receptor-γ coactivator (*PGC1α*, *p* < 0.05), which is known to facilitate mitochondrial fatty acid oxidation and hence lipolysis (Figure 2E). SIRT1 expression in Epi WAT was not changed by either maternal or paternal SIRT1 overexpression, although it showed a trend of recovering with the former approach. *SREBP1c* expression, on the other hand, was partially reduced by maternal but not paternal SIRT1 overexpression (Figure 2E).

Similar to Epi WAT, Rp WAT also caused reduced *SIRT1* expression (*p* < 0.05, Supplementary Figure S2). However, in contrast to the upregulation of *SREBP-1c*, *PPARγ* and *PGC1α* in Epi WAT, the expression of these markers in Rp WAT was significantly lower in MHF offspring compared to the control (*p* < 0.05), which was significantly reversed by maternal (but not paternal) SIRT1 overexpression (*p* < 0.05). Paternal SIRT1 overexpression significantly improved the expression of *PGC1α* (*p* < 0.05), and showed trends towards normalising the expressions of *SREBP-1c* and *PPARγ*.

### 2.4. Parental SIRT1 Overexpression Attenuated Liver Lipogenesis Markers but not Steatosis in MHF Offspring

**Figure 3:**
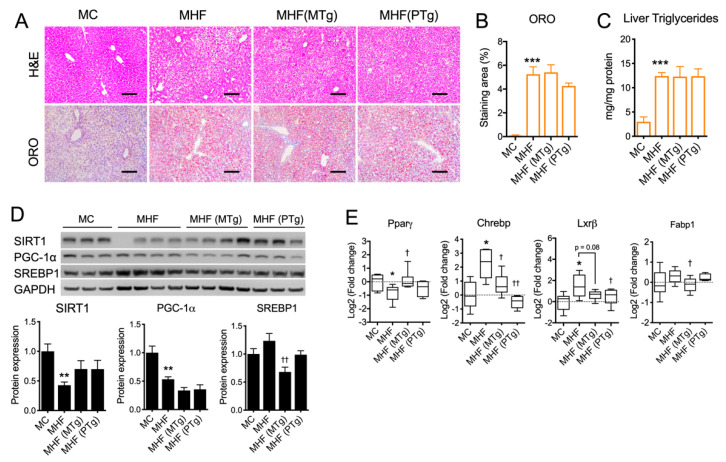
Effects of parental SIRT1 overexpression on hepatic morphology and the regulation of metabolic markers in MHF offsprings’ livers (**A**) H&E and Oil Red O staining. Scale bar = 100 μm. (**B**) Oil Red O staining quantitation. (**C**) Liver triglyceride level. (**D**,**E**) protein and mRNA expression of metabolic markers. *n* = 4–6. vs. MC: * *p* < 0.05, ** *p* < 0.01, *** *p* < 0.001; vs. MHF: † *p* < 0.05, †† *p* < 0.01.

Hepatocyte cytoplasmic vacuolation, an indicator of glycogen accumulation, was evident in MHF offspring (Figure 3A). Lipid droplet accumulation, as per Oil Red O (ORO) staining quantitation, as well as the level of triglyceride were both significantly increased in the offspring’s liver as a result of maternal HFD consumption (*p* < 0.001, Figure 3A–C). Such impacts on hepatic morphology were associated with significant reductions in the hepatic protein expression of SIRT1 and PGC-1α (*p* < 0.01, Figure 3D), which were not relieved by either maternal or paternal SIRT1 overexpression. Lipogenic marker SREBP1 showed a trend towards being increase in the MHF offspring’s liver, which was significantly downregulated by maternal SIRT1 overexpression (*p* < 0.05, Figure 3D). Similarly, the mRNA expression of Carbohydrate-response element-binding protein (*CHREBP*) and liver X receptor beta (*LXRβ*) was significantly increased (*p* < 0.05, Figure 3E). Maternal SIRT1 overexpression significantly reduced the levels of Chrebp (*p* < 0.05) and Fabp1 (*p* < 0.05). It also showed a trend towards downregulating *LXRβ* expression (*p* = 0.08). Paternal SIRT1 suppressed *CHREBP* and *LXRβ* expression (*p* < 0.01 and *p* < 0.05 respectively). Despite the suppression of lipogenic markers, SIRT1 overexpression in dams or sires did not significantly improve hepatic morphology or lower steatosis in MHF offspring (Figure 3A–C).

Further examination of AMP kinase (AMPK), an important regulator of multiple cellular metabolic processes including glycolysis and lipolysis, indicated the downregulation of both AMPK protein expression (*p* < 0.05) and its activity (reflected by the level of phosphorylated AMPK or pAMPK, *p* < 0.05, Supplementary Figure S3A). Similarly, the protein expression of Protein kinase B (Akt), a master regulator of glycolysis and cell survival, and its activity reflected by the level of phosphorylated Akt (pAkt) were both significantly suppressed (*p* < 0.05 and *p* < 0.01, respectively, Supplementary Figure S2A). Maternal SIRT1 overexpression significantly improved pAMPK/AMPK ratio (*p* < 0.05), but had no effect on Akt expression/activity. In comparison, paternal SIRT1 overexpression increased total Akt protein expression (*p* < 0.05), but conversely suppressed its activity (*p* < 0.01). Regarding the regulation of glucose mobilisation, the mRNA expressions of glucose transporters (*GLUT*)1 and 2 were both significantly suppressed (*p* < 0.05 and *p* < 0.001, respectively, Supplementary Figure S3B). Consistently, the protein expression of GLUT2 was also reduced (*p* < 0.01). Parental SIRT1 overexpression had no effect on the expression of these glucose transporters.

### 2.5. Parental SIRT1 Overexpression Suppressed Hepatic Inflammation in MHF Offspring

**Figure 4:**
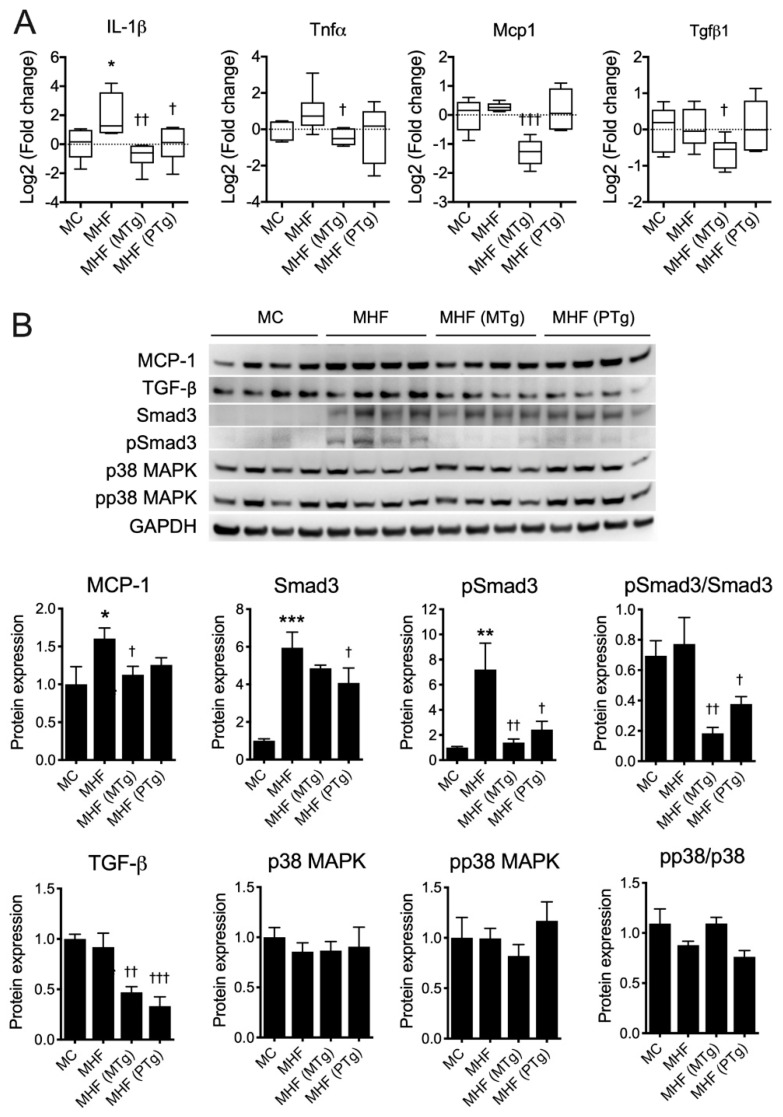
Parental SIRT1 overexpression attenuates inflammation in the MHF offspring’s liver. (**A**) mRNA expression and (**B**) protein expression of inflammatory markers (*n* = 4–6) vs. MC: * *p* < 0.05, ** *p* < 0.01, *** *p* < 0.001; vs. MHF: † *p* < 0.05, †† *p* < 0.01, ††† *p* < 0.001.

Interleukin (IL)-1β mRNA expression was significantly increased in the MHF offspring’s liver (*p* < 0.05, Figure 4A). Other proinflammatory cytokines, including Tumour Necrosis Factor alpha (*TNFα*), showed a tendency to elevate, while monocyte chemoattractant protein (*MCP1*) and transforming growth factor beta (*TGFβ*)-1 showed no changes at the transcriptional levels. Maternal SIRT1 overexpression significantly suppressed the mRNA levels of *IL-1β* (*p* < 0.01), *TNFα* (*p* < 0.05), *MCP1* (*p* < 0.001) and *TGFβ1* (*p* < 0.05). Paternal SIRT1 overexpression only induced suppression on IL-1β (*p* < 0.05).

Consistent with the RT-qPCR results, our immunoblot data indicated an elevation of MCP-1 protein expression in offspring liver due to maternal HFD consumption (*p* < 0.05, Figure 4B), which was reversed by maternal SIRT1 overexpression (*p* < 0.05). The protein expression of TGFβ1 was unchanged, but that of its downstream factor Smad3 was markedly increased in the MHF offspring’s liver (*p* <0.001). Moreover, the level of phosphorylated Smad3 (pSmad3) was also significantly upregulated (*p* < 0.01), suggesting a higher level of activation of the TGFβ-Smad pathway. There were no changes in the regulation of the TGFβ non-Smad pathway as reflected by the unchanged levels of p38 mitogen-activated protein kinase (MAPK) and its phosphorylated isoform (*p*-p38). Both maternal and paternal SIRT1 overexpression significantly reduced the hepatic protein expression of TGF-β1 in offspring born to dams fed the HFD (*p* < 0.01 and *p* < 0.001, respectively). Consistently, both interventions also suppressed the level of *p*-Smad3 (*p* < 0.01 and *p* < 0.05, respectively) and the p-Smad3/Smad3 ratio (*p* < 0.01 and *p* < 0.05, respectively), suggesting the attenuated activation of this pathway. No changes were found in MHF offspring born to Tg parents in comparison to MHF offspring born to WT parents, with regard to the TGFβ-p38 signalling axis.

Regarding oxidative stress, the mRNA expression of NAPDH oxidase 2 (*NOX2*) showed a trend to increase, while that of superoxide dismutase (*SOD*)2 was significantly reduced in the liver of MHF offspring (*p* < 0.05 and *p* < 0.01, respectively, Supplementary Figure S4). Glutathione Peroxidase (Gpx)-1 also showed a tendency to reduce, while the level of Catalase (*CAT*) was unchanged in the MHF offspring’s liver. Maternal and paternal SIRT1 overexpression had no effects on any examined markers except CAT, in which both interventions led to the significant upregulation of this marker (*p* < 0.05), suggesting a modest improvement in antioxidant capacity.

### 2.6. Parental SIRT1 Overexpression Suppressed Global Methylation in MHF Offspring Liver

**Figure 5:**
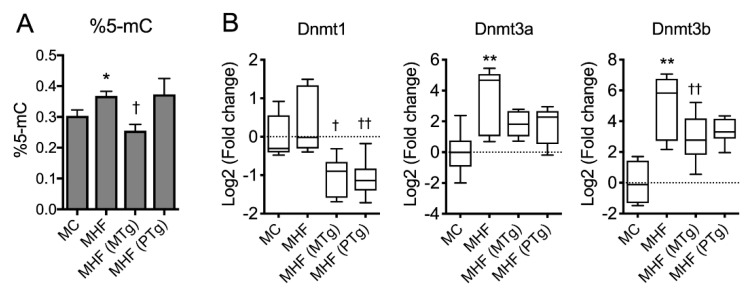
Parental SIRT1 overexpression suppressed global methylation in MHF offspring liver. (**A**) Global DNA methylation. (**B**) mRNA expression of DNA methyltransferases (DNMT). *n* = 4–6. vs. MC: * *p* < 0.05, ** *p* < 0.01; vs. MHF: † *p* < 0.05, †† *p* < 0.01.

Maternal HFD consumption was associated with a significant increase in global methylation, reflected by the percentage of methylated cytosine (%5-mC) in the offspring’s liver (*p* < 0.05, Figure 5A). Consistently, the mRNA expression of DNA methylation transferase Dnmt1 also showed a trend to increase, and those of Dnmt3a and Dnmt3b were significantly increased in MHF offspring compared to MC offspring (*p* < 0.01, Figure 5B). Maternal SIRT1 overexpression significantly suppressed global DNA methylation (*p* < 0.05) as well as mRNA expression of Dnmt1 and Dnmt3b (*p* < 0.001 and *p* < 0.05, respectively). Paternal SIRT1 overexpression also significantly suppressed the expression of Dnmt1 (*p* < 0.01), but did not affect Dnmt3b or global DNA methylation levels. Dnmt3a showed a trend to be suppressed by both maternal and paternal SIRT1 overexpression (*p* = 0.09).

## 3. Discussion

Consistent with previous studies, our study demonstrates that maternal HFD consumption increased body weight, adiposity, glucose intolerance and insulin resistance in the offspring. In addition, adipocyte morphology and lipid metabolism were impaired, and the liver showed increased levels of steatosis, inflammation and oxidative stress. These changes were found to be associated with the reduced expression of SIRT1 in the offspring’s WAT and liver. Increasing SIRT1 expression not only reduced body weight and adiposity in the dams and sires per se, but also prevented the increase in body weight, adiposity, hyperlipidaemia and adipocyte overgrowth in the offspring due to maternal HFD consumption. Maternal SIRT1 overexpression in particular also improved glucose tolerance and increased blood leptin levels, while suppressing milk intake and improving adipocyte morphology. Both maternal and paternal SIRT1 overexpression ameliorated lipid dysregulation in the WAT and liver, and also lowered the hepatic abundance of proinflammatory cytokines in offspring born to HFD-fed dams. Interestingly, such effects were associated with reduced levels of DNA methylation markers.

The direct role of SIRT1 in regulating the metabolism is well-established [16]. Previous studies have shown that increasing SIRT1 expression or activity can mitigate obesity and related disorders, including adiposity, insulin resistance and hepatic steatosis [17,18]. However, few have examined the effect of SIRT1 across generations [19]. Our study demonstrated that SIRT1 overexpression not only restricted HFD-induced weight gain and adiposity in female mice at conception and throughout perinatal periods, but also led to a leaner phenotype in their offspring, despite no significant changes in maternal blood glucose or lipid levels. This result implies an optimisation of nutrient supply via placental or milk, and/or improved metabolism in the foetuses/neonates, which requires further investigation of placental nutrient transportation and milk quality. It was previously shown that maternal obesity caused the upregulation of glucose and fatty acid transporters in association with reduced SIRT1 expression in the placenta. In this study, placental examination was not possible, as placentas were consumed by dams immediately after giving birth. In order to measure milk consumption, offspring were briefly fasted and then refed. The body weight difference between the two phases was used to estimate their milk consumption. The data demonstrated a delayed recovery in body weight in offspring born to transgenic dams after refeeding, suggesting either reduced milk production by the dams, reduced suckling by the offspring or both. This change was associated with a higher level of circulating leptin, an appetite-suppressing hormone released by adipose tissue, which acts on leptin receptors in the hypothalamus. It was also associated with the normalised expression of appetite-regulating neuropeptides Npy and Pomc in the offspring’s hypothalamus. Together, the data support the effect of maternal SIRT1 overexpression in improving MHF offspring’s appetite regulation. Paternal SIRT1 overexpression, on the other hand, did not alter milk intake or Npy/Pomc levels, but significantly increased hypothalamic expression of SIRT1 and Ob-Rb. This is consistent with a previous study that showed increased leptin signalling in hypothalamus-specific SIRT1-overexpressing mice [20].

Consistent with previous studies, maternal HFD consumption induced glucose intolerance and hyperlipidaemia in offspring, which were associated with accelerated adipocyte growth and altered adipocyte morphology in Epi WAT. Concomitantly, SIRT1 expression in offspring Epi and Rp WAT was downregulated. Both maternal and paternal SIRT1 overexpression substantially reduced adiposity and hyperlipidaemia, but only the former improved glucose tolerance and adipocyte morphology in MHF offspring. Such changes in offspring born to transgenic dams aligned with a partial recovery in SIRT1 expression and the repression of SREBP-1c in their Epi WAT, as well as a complete normalisation of SIRT1, SREBP-1c, PPARγ and PGC-1α in Rp WAT. In comparison, paternal SIRT1 overexpression had modest effects on the regulation of these markers, especially in Epi WAT. These results suggest that increasing SIRT1 expression in mothers is likely to improve SIRT1 signalling and reverse the dysregulation of lipid metabolism in the offspring’s visceral fat, which in turn reduces the risk of systemic dyslipidaemia and type 2 diabetes. Of note, the expression of SREBP-1c, PPARγ and PGC-1α was differently regulated in Epi and Rp WAT, a phenomenon that had been reported previously by our group in the same model [9].

Maternal obesity has been associated with the development of non-alcoholic fatty liver disease (NAFLD) in offspring, characterised by an increase in lipid droplet accumulation and inflammation in the liver [21,22], which is also what we found in the current study. SIRT1 expression is reduced in the offspring’s liver in association with the downregulation of lipolytic markers, such as PGC-1α and AMPK, as well as the upregulation of lipogenic markers ChREBP and LXRβ, which may underline the increased lipid accumulation. Both AMPK and Akt are known to stimulate glycolysis and promote cell survival during nutritional stress, thus their reduction in the MHF offspring’s liver indicates a state of overnutrition. The concomitant decreases of hepatic GLUT1 and 2 also support a reduction in glucose mobilisation and utilisation in the MHF offspring, and are consistent with increased glycogen storage, as reflected by hepatocyte vacuolation. PPARγ is a special case as it acts both as an insulin sensitiser and adipogenic/lipogenic marker [23], thus the reductions of its expression in not only the liver but also Rp WAT in association with glucose intolerance and insulin resistance signals that its primary function leans toward promoting glucose homeostasis at this age [24]. Supporting this hypothesis, maternal SIRT1 overexpression significantly improved PPARγ expression in the liver and Rp WAT, and reversed glucose tolerance in MHF offspring. Maternal SIRT1 overexpression also improved AMPK activation, while suppressing SREBP-1c, ChREBP and FABP1, and paternal SIRT1 overexpression downregulated ChREBP and LXRβ. Neither maternal or paternal SIRT1 overexpression had an effect on the activation of Akt or the expression of GLUTs, suggesting that the effects of parental SIRT1 expression were independent of Akt signalling and the regulation of glucose transport in the offspring’s liver. Although these molecular changes were insufficient to rescue hepatic morphology, they likely had positive impacts on inflammation. Indeed, offspring born to transgenic dams showed lower hepatic expression of IL-1β, TNF-α and MCP-1, while those born to transgenic sires had lower levels of IL-1β. Both intervention approaches suppressed TGF-β and downstream Smad signalling.

The mechanisms whereby parental SIRT1 overexpression influences SIRT1 expression and related pathways in the offspring are unclear. However, several mechanisms can be proposed. Regarding the maternal effect, apart from the improved intrauterine environment that is likely to rescue the foetal programming effects of maternal obesity, SIRT1 overexpression in dams is likely to include increased SIRT1 expression in zygotes. This provides an immediate benefit to foetal metabolism. Supporting this hypothesis, it has been shown that Resveratrol, an activator of SIRT1, was able to alleviate obesity-induced oxidative stress and growth retardation in oocytes [25]. As SIRT1 is a histone acetylase, epigenetic modification may also contribute to this programming process. Indeed, the fact that paternal SIRT1 overexpression was able to increase SIRT1 expression in the offspring’s hypothalamus and Rp WAT, without changes in the intrauterine environment or the offspring’s genotype, is likely to reflect epigenetic regulation, for instance via sperm-derived microRNAs. Although this hypothesis needs to be validated by further studies, supporting evidence can be found in other models of chronic stress. For example, in a mouse model of chronic social defeat stress, the microinjection of paternal stress-related sperm microRNAs into zygotes led to the marked depletion of SIRT1 mRNA, which was associated with hypothalamic–pituitary–adrenal stress dysregulation in the offspring [3]. In our model, an increase in paternal SIRT1 expression may confer an elevation of beneficial microRNAs that counteract the decrease in SIRT1 in zygotes due to maternal HFD consumption. Besides, SIRT1 has been shown to play a pivotal role in spermatogenesis and germ-cell function [26].

DNA methylation is the most studied aspect of epigenetic programming. Particularly regarding maternal obesity, human studies using cord blood found that global DNA methylation is higher in newborns with higher birthweights compared with controls. This was associated with increased adiposity in later life [27]. In this study, by demonstrating that paternal SIRT1 overexpression can suppress DNA methylation markers in MHF offspring, we provide supporting data to suggest that DNA methylation is likely to be one of the pathways whereby parental SIRT1 regulates metabolic signature across generations. Although both maternal and paternal SIRT1 overexpression downregulated DNMT1, only the former significantly suppressed global DNA methylation. This is likely due to the effect of maternal SIRT1 overexpression co-suppressing DNMT3a and DNMT3b, which are essential for de novo DNA methylation. Further studies are needed to identify specific genes that are differentially methylated in the offspring’s liver due to maternal HFD consumption, and to investigate whether parental SIRT1 overexpression normalises these changes.

In this study, we did not investigate offspring in adulthood to see if parental SIRT1 therapy has long-lasting effects on the offspring’s metabolism. However, according to our previous studies, the effects of maternal obesity tend to be gradually overpowered by the effects of postnatal diet at later time points [28]. This is likely because epigenetic modifications are somewhat reversible, and lifestyle modification, including dietary changes, is considered one of the strongest modifiable factors influencing metabolic health. Thus, the aim of early interventions during pregnancy is rather to offer a healthier kickstart than to ensure life-long benefits in the offspring.

Collectively, our study suggests that increasing SIRT1 expression in either parent can attenuate the metabolic and liver disorders induced in offspring by maternal high-fat diet feeding. Intervention through paternal SIRT1 overexpression is novel, and particularly useful as it does not raise concerns about the possible side effects of SIRT1 therapy during gestation and early childhood. Further studies are required to investigate the long-term effects of both maternal and paternal SIRT1 therapy in the offspring.

## 4. Materials and Methods

### 4.1. Animals

The study was approved by the Animal Care and Ethics Committee of the University of Sydney and Royal North Shore Hospital, Australia (RESP/15/22). All animal experiments were performed at the Kearns Facility, Kolling Institute, Royal North Shore Hospital in accordance with the relevant guidelines and regulations in the Australian Code of Practice for the Care and Use of Animals for Scientific Purposes. Female C57BL/6 mice (8 weeks) or SIRT1-Tg mice were fed a HFD (20 kJ/g, 43.5% calorie as fat, Specialty Feed, WA, Australia) or standard rodent chow (11 kJ/g, 14% calorie as fat, Gordon’s Speciality Stockfeeds, NSW, Australia) for 6 weeks before mating, throughout gestation and lactation [29]. Hemizygous Tg dams or sires were crossed with wild-type breeders to generate both WT and Tg offspring. However, only WT male offspring were selected for the study to normalise genetic differences and because the effects of maternal obesity-induced foetal programming appear to be more prominent in males [19]. Newborns were genotyped at postnatal (*p*)14 as previously described [9]. To limit the difference in milk competition, litter size was adjusted to an average of five ± one pups per dam. Four experimental groups were included: (1) MC—Chow-fed WT-dam × Chow-fed WT-sire; (2) MHF—HFD-fed WT-dam × Chow-fed WT-sire; (3) MHF-MTg—HFD-fed hemizygous Tg-dam × Chow-fed WT-sire; and (4) MHF-PTg—HFD-fed WT dam × Chow-fed Tg-sire. The Tg mouse colony was acquired from Dr Lindsay Wu (University of New South Wales, Sydney, NSW, Australia) [30].

### 4.2. Milk Intake Estimation

Offspring were separated from their dams at postnatal day (*p*)16 and fasted for 4 h. They were weighed before returning to their dams. After 2 h of feeding, the offspring were weighed again. The difference in the body weight during the 2 h period was used for milk intake estimation. No access to solid food was provided during the lactation phase to ensure no chow/HFD consumption by the pups.

### 4.3. Intraperitoneal Glucose Tolerance Test (IPGTT)

At postnatal day (*p*)18, the animals were weighed and fasted for 4 h prior to IPGTT, then a glucose solution (50%) was injected (2 g/kg, i.*p*.). Tail blood glucose level (BGL) was recorded prior to glucose injection at 15, 30, 60 and 90 min post-injection using a glucometer (Accu-Chek glucose meter; Roche Diagnostics). The area under the curve (AUC) for glucose was calculated for each animal.

### 4.4. Tissue Collection

At weaning (P20), all pups were sacrificed after 4 h of fasting. Blood was collected via cardiac puncture under anaesthesia (3% isofluorane and 1% oxygen). Phosphate-buffered saline (PBS, 1%) was used for whole body perfusion. Tissues, including Epi and Rp WAT, hypothalamus and liver, were snap frozen and stored at −80 °C or fixed in neutral buffered formalin (10%) for approximately 36 h for later analyses.

### 4.5. Protein and Lipid Extraction

The tissues were homogenised in Triton X-100 lysis buffer (pH 7.4, 150 mM NaOH, 50 mM Tris-HCl, 1% Triton X-100, Roche protease inhibitor) using TissueRuptor (Qiagen, Hilden, Germany). Lipid and protein were extracted and measured for triglyceride concentrations according to our previously published protocol [19] using Roche triglyceride reagent GPO-PAP (Roche Life Science, NSW, Australia) and Pierce BCA Protein Assay Kit (Thermo Scientific, VIC, Australia) according to the manufacturer’s instructions. Lipid concentrations were normalised to protein concentration from the same extraction.

### 4.6. Blood Insulin, Free Fatty Acid and Triglyceride Assay

Blood was collected in a K2EDTA blood collection tube (Becton Dickinson, NSW, Australia) then centrifuged at 1500× *g* at 4 °C for 10 min. Plasma (supernatant) was carefully removed into a new tube and stored at −20 °C until biochemical assessment. The plasma insulin level was measured by ELISA (Abcam, Cambridge, UK). The homeostatic model assessment for insulin resistance (HOMA-IR) score was calculated using the following formula: fasting plasma glucose (mmol/l) times fasting serum insulin (mU/L) divided by 22.5. The levels of non-esterified fatty acid (NEFA) and triglyceride were measured using HR Series NEFA-HR (FUJIFILM Wako Diagnostics, CA, USA) and GPO-PAP reagents (Roche Life Science, NSW, Australia), respectively.

### 4.7. Histological Analysis

Tissues were fixed in 10% formalin for 36 h and embedded in paraffin or frozen-embedded in OCT solution (Tissue-Tek). Paraffin sections and frozen sections were prepared at 4 μm and 12 μm thickness, respectively, and mounted on microscope slides (Trajan Scientific and Medical, VIC, Australia). Ep WAT and liver were stained with Hematoxylin and Eosin (H&E) for histological analysis. For each sample, six random non-overlapping fields were captured at 200× magnification using a bright-field microscope (Leica Microsystems, Wetzlar, Germany). Adipocyte size was measured using Adiposoft, a plugin of the Image J software (https://imagej.net/Adiposoft, January 2019). Adipose tissue morphology was graded from 0 to 4 based on the occurrence of multilocular adipocytes [31], which reflects the ‘beiging’ level of WAT. The score 0 represents 100% adipocytes being mature with 1 single lipid droplet (unilocular), 1 represents up to 25% of adipocytes being multilocular, 2 represents a 25–50% occurrence of multilocular adipocytes, 3 represents extensive adipose tissue remodeling with 50–75% of adipocytes being multilocular, and 4 indicates that the majority of adipocytes (>75%) are multilocular. Slides were coded and assessed blindly to ensure objectivity. For lipid droplet visualisation in liver tissues, Oil Red O (ORO) staining was used as previously described [9].

### 4.8. Quantitative RT-PCR

The total RNA of liver tissues was isolated using RNeasy Plus Mini Kit (Qiagen, Hilden, Germany) according to the manufacturer’s instructions, while RNA from fat tissue was extracted using Trizol Reagent (Sigma-Aldrich). The purified total RNA was used as a template to generate the first-strand cDNA using the First Strand cDNA Synthesis Kit (Roche Life Science, NSW, Australia). The amplicons of target genes were amplified with SYBR Green probes. Primer sequences are summarised in Supplementary Table S1. Gene expression was standardised to β-actin mRNA and log-transformed. Before acquiring the actual data, all of the new primers were tested for amplification efficiency (90–110%) and specificity (single peak in dissociation curve analysis). The final concentration for all primers in a quantitative PCR reaction was 200 nM. Several commonly used housekeeping genes, including 18s, α-tubulin and β-actin, were tested. B-actin showed the least variation in mRNA expression among the groups. Therefore, gene expression was standardised to β-actin mRNA and log-transformed.

### 4.9. Immunoblotting

Protein samples were loaded in equal amounts on Bolt 4–12% Bis-Tris Plus Gels (Life Technologies, VIC, Australia) for protein electrophoresis. The gel was transferred onto a Hybond nitrocellulose membrane (Amersham Pharmacia Biotech, Amersham, UK), which was blocked with 5% skim milk and incubated with different primary antibodies at 4 °C overnight, followed by washing and incubation with secondary antibodies for 1 h at room temperature. The immunoblots were developed by adding the Luminata Western HRP Substrates (Millipore, MA, USA) to the membrane and exposed for an appropriate duration using ImageQuant LAS 4000 (Fujifilm, Tokyo, Japan). ImageJ (National Institutes of Health, USA) was used for densitometric analyses. The antibodies’ information and concentrations were summarised in Supplementary Table S2. GAPDH was used for loading control.

### 4.10. Global DNA Methylation Analysis

DNA was extracted from offspring livers using a DNeasy Blood and Tissue Kit (Qiagen, Hilden, Germany). DNA purity was tested by NanoDrop 1000 Spectrophotometer (Thermo Fisher Scientific, MS, USA). The level of global DNA methylation level was measured in the offspring liver as percentage of methylated cytosine (%5-mC) using the MethylFlash Global DNA Methylation ELISA Easy Kit (Epigentek, NY, USA) according to the manufacturer’s instructions.

### 4.11. Statistical Analysis

To confirm the effects of maternal HFD consumption on the offspring, unpaired *t*-test was used to compare MC and MHF groups. To address our main hypothesis that maternal or paternal SIRT1 overexpression can attenuate maternal obesity-induced metabolic disorders in the offspring, one-way ANOVA followed by Fisher’s LSD comparison was used for statistical analysis between MHF and the two intervention groups. PCR data are presented as box plots that extend from the 25th to 75th percentiles, with the central lines indicating the median and whiskers ranging from Minimum to Maximum. Other data are presented as mean ± SEM.

## Figures and Tables

**Table 1:** Anthropometric and metabolic changes in the offspring.

Group	MC	MHF	MHF (MTg)	MHF (PTg)
*n*	16	20	12	23
BW	8.26	11.59 ***	9.33 †††	9.87 †††
SEM	0.04	0.04	0.09	0.06
EpiWAT (g)	0.024	0.131 ***	0.073 †††	0.092 †††
SEM	0.000	0.002	0.002	0.001
EpiWAT (%BW)	0.292	1.120 ***	0.771 †††	0.920 ††
SEM	0.004	0.012	0.021	0.007
RpWAT (g)	0.0061	0.0289 ***	0.0182 ††	0.0277
SEM	0.0001	0.0003	0.0005	0.0004
RpWAT (%BW)	0.073	0.249 ***	0.195 †	0.280
SEM	0.002	0.003	0.005	0.004
Liver (g)	0.400	0.648 ***	0.521 ††	0.479 †††
SEM	0.004	0.005	0.012	0.004
Liver (%BW)	4.846	5.552 **	5.489	4.833 ††
SEM	0.050	0.032	0.085	0.029
Milk intake (g)	0.036	0.134 **	0.042 ††	0.095
SEM	0.005	0.005	0.008	0.004
Blood leptin level	0.998	2.545 *	4.476 †	3.667
SEM	0.042	0.211	0.246	0.453
Fasting BGL (mM)	10.26	13.31 ***	12.64	13.27
SEM	0.09	0.15	0.28	0.06
IPGTT AUC	957	1636 ***	1214 †	1412
SEM	17	89	28	77
Fasting insulin level (μIU/mL)	17.40	27.03 *	24.58	24.74
SEM	0.85	1.02	1.38	0.64
HOMA-IR	7.72	15.35 **	14.49	14.45
SEM	0.30	0.86	1.05	0.86
Blood triglyceride level (μM)	0.303	1.392 ***	0.775 †	0.771 †
SEM	0.014	0.092	0.090	0.082
Blood NEFA level (μM)	0.272	0.944 ***	0.519 †††	0.439 †††
SEM	0.006	0.026	0.031	0.012

vs. MC: * *p* < 0.05, ** *p* < 0.01, *** *p* < 0.001; vs. MHF: † *p* < 0.05, †† *p* < 0.01, ††† *p* < 0.001.

## Supplementary Materials

Supplementary Materials can be found at https://www.mdpi.com/1422-0067/21/19/7342/s1.

## Abbreviations

ACEC	Animal Care and Ethics Committee
Akt	Protein kinase B
AMPK	AMP kinase
AUC	Area under the curve
BGL	Blood glucose level
CAT	Catalase
ChREBP	Carbohydrate response-element-binding protein
DNMT	DNA methyltransferase
Epi	Epididymal
FABP	Fatty acid binding protein
GLUT	Glucose transporter
GPO-PAP	glycerine phosphate oxidase peroxidase
GPX	Glutathione peroxidase
HFD	High-fat diet
HOMA-IR	Homeostatic Model Assessment of Insulin Resistance
IPGTT	Intraperitoneal glucose tolerance test
MCP-1	Macrophage chemotactic protein 1
MHF	Maternal high-fat diet consumption
MTg	Maternal SIRT1 overexpression
NOX	NADPH oxidase
NPY	Neuropeptide Y
Ob-Rb	Leptin receptor
PGC-1α	Peroxisome proliferator-activated receptor gamma coactivator 1-α
POMC	Proopiomelanocortin
PPAR	Peroxisome proliferator-activated receptors
PTg	Paternal SIRT1 overexpression
ROS	Reactive oxygen species
Rp	Retroperitoneal
SIRT	Silent information regulator
SOD	Superoxide dismutase
SREBP	Acetylated sterol regulatory element binding proteins
Tg	Transgenic
TGF-β	Transforming growth factor beta
TNFα	Tumour necrosis factor alpha
WAT	White adipose tissue
WT	Wildtype
