# Supplementary material for: Parental SIRT1 Overexpression Attenuate Metabolic Disorders Due to Maternal High-Fat Feeding
Source: Int J Mol Sci. 2020 Oct 5;21(19):7342. doi: 10.3390/ijms21197342 (PMC7582993; doi:10.3390/ijms21197342)

**Supplementary info**

**Table S1. Primer sequences**

| No. | Gene | Forward primer sequence | Reverse primer sequence |
|---|---|---|---|
| 1 | Actin | CTAAGGCCAACCGTGAAAAG | ACCAGAGGCATACAGGGACA |
| 2 | Cat | CTCCATCAGGTTTGTTTCTTG | CAACAGGCAAGTTTTTGATG |
| 3 | Srebp-1c | GGAGCCATGGATTGCACATT | GCTTCCAGAGAGGAGGCCAG |
| 4 | Chrebp | ATATCTCCGACACACTCTTC | CAACATAAGCATCTTCTGGG |
| 5 | Fabp1 | CACGGACTTTATGCCTTTGAA | GACCTCATCCAGAAAGGGAAG |
| 6 | Glut2 | TCGCCCTCTGCTTCCAGTAC | GAACACGTAAGGCCAAGGA |
| 7 | Gpx1 | GGACAATGGCAAGAATGAAG | TTCGCACTTCTCAAACAATG |
| 8 | Lxrb | TCACCCACTATTAAGGAAGAG | TCTAAGATGACCACGATGTAG |
| 9 | Npy | GGCTGTGTGGACTGACCCT | GATGTAGTGTCGCAGAGCGG |
| 10 | Nox2 | CTACCTAAGATAGCAGTTGATG | TACCAGACAGACTTGAGAATG |
| 11 | Ob-rb | CCAGGTGAGGAGCAAGAG | CTGCACAGTGCTTCCCAC |
| 12 | Pgc1a | AAACTTGCTAGCGGTCCTCA | TGGCTGGTGCCAGTAAGAG |
| 13 | Pomc | GAGATTCTGCTACAGTCGCTC | TTGATGATGGCGTTCTTGAA |
| 14 | Pparg | ATCTACACGATGCTGGC | GGATGTCCTCGATGGG |
| 15 | Sirt1 | GCAGGTTGCGGGAATCCAA | GGCAAGATGCTGTTGCAAA |
| 16 | Sod1 | CACTCTAAGAAACATGGTGG | GATCACACGATCTTCAATGG |
| 17 | Sod2 | GGCCTACGTGAACAACCTGAA | CTGTAACATCTCCCTTGGCCA |
| 18 | Tnfa | CTGTAGCCCACGTCGTAGC | TTGAGATCCATGCCGTTG |
| 19 | Dnmt1 | GTGAACAGGAAGATGACAAC | CTGGATCCTCCTTTCATTC |
| 20 | Dnmt3a | ACCAGAAGAAGAGAAGAATCC | CAATGATCTCCTTGACCTTAG |
| 21 | Dnmt3b | GACTTCATGGAAGAAGTGAC | TATCATCCTGATACTCTGTGC |

**Table S2. Antibody information**

| Target | Host | Size (kDa) | Dilution (WB) | Cat# | Company | Address |
|---|---|---|---|---|---|---|
| GAPDH | Mouse | 38 | 1:2000 | ab8245 | Abcam | Cambridge, UK |
| SIRT1 | Rabbit | 110 | 1:2000 | 07-131 | EMD Millipore | NSW, Australia |
| PGC-1α | Rabbit | 91 | 1:2000 | NBP1-04676 | Novus Biologicals | CO, USA |
| SREBP1 | Mouse | 62 | 1:500 | ab3259 | Abcam | Cambridge, UK |
| GLUT2 | Rabbit | 50 | 1:1000 | 07-1402-I | EMD Millipore | NSW, Australia |
| p-Akt (Ser473) | Rabbit | 60 | 1:1000 | 9271 | Cell Signalling | MA, USA |
| Akt | Rabbit | 60 | 1:1000 | 9272 | Cell Signalling | MA, USA |
| p-AMPKα (Thr172) | Rabbit | 62 | 1:1000 | 2535 | Cell Signalling | MA, USA |
| AMPKα | Rabbit | 62 | 1:1000 | 2532 | Cell Signalling | MA, USA |
| MCP1 | Rabbit | 25 | 1:2000 | ab8101 | Abcam | Cambridge, UK |
| TGF-β | Rabbit | 27 | 1:1000 | 3711 | Cell Signalling | MA, USA |
| p-Smad3 (Ser423/425) | Rabbit | 55 | 1:500 | 9513 | Cell Signalling | MA, USA |
| Smad3 | Rabbit | 55 | 1:1000 | 9520 | Cell Signalling | MA, USA |
| p-p38 MAPK (Thr180/Tyr182) | Rabbit | 38 | 1:1000 | 4511 | Cell Signalling | MA, USA |
| p38 MAPK | Rabbit | 38 | 1:1000 | 8690 | Cell Signalling | MA, USA |

**Figure S1.**

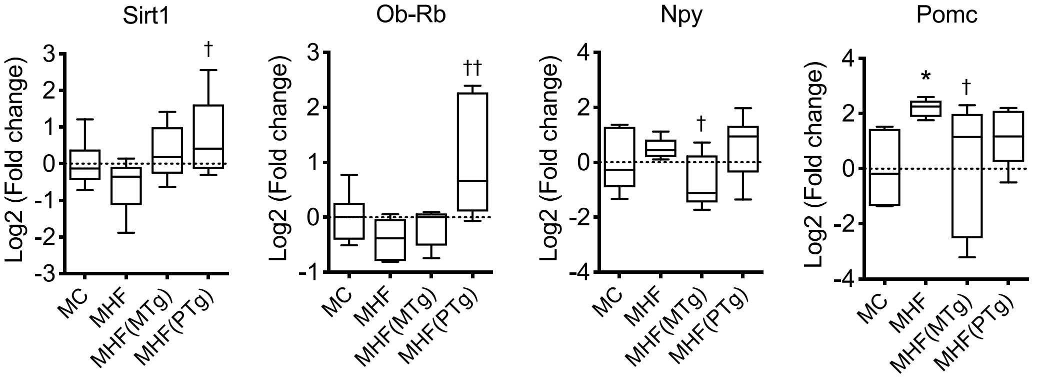

**Figure S2.**

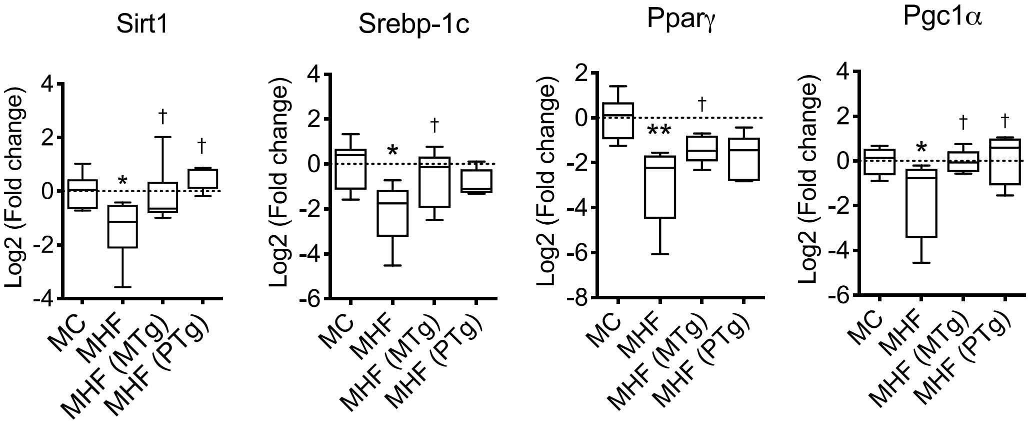

Sirt1
Srebp-1c
Pparγ
Pgc1α
Log2 (Fold change)
MC
MHF
MHF (MTg)
MHF (PTg)

**Figure S3.**

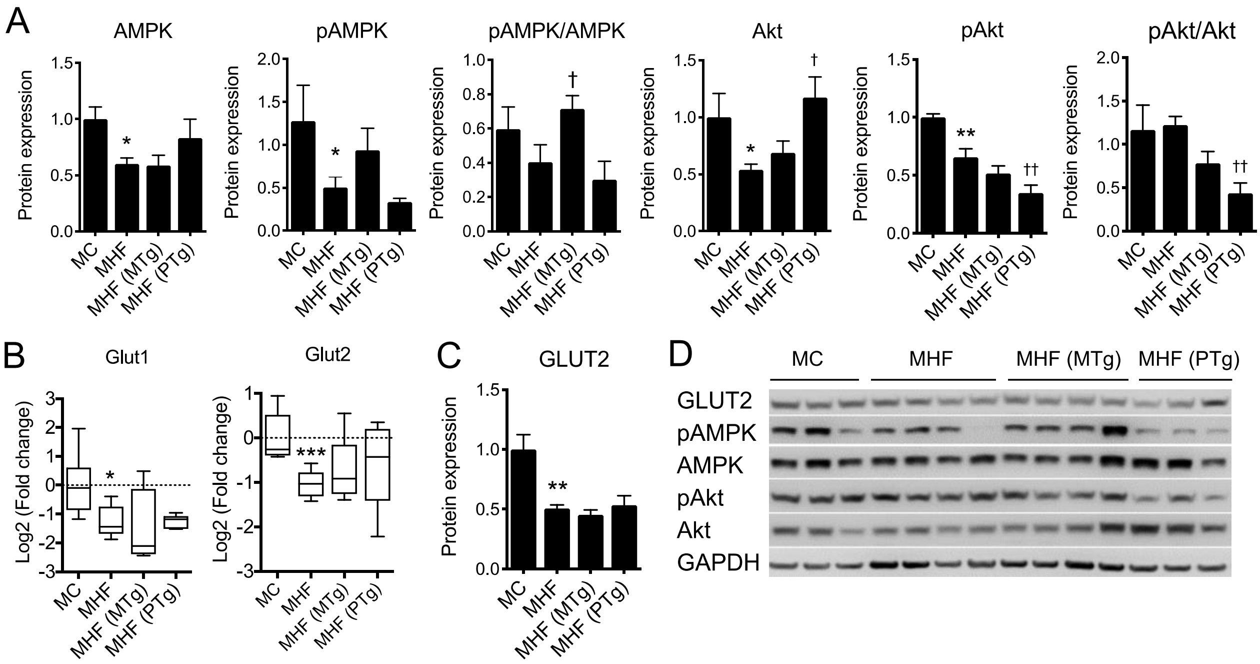

A
AMPK
pAMPK
pAMPK/AMPK
Akt
pAkt
pAkt/Akt
Protein expression
MC
MHF
MHF (MTg)
MHF (PTg)
B
Glut1
Glut2
Log2 (Fold change)
C
GLUT2
D
MC
MHF
MHF (MTg)
MHF (PTg)
GLUT2
pAMPK
AMPK
pAkt
Akt
GAPDH

**Figure S4.**

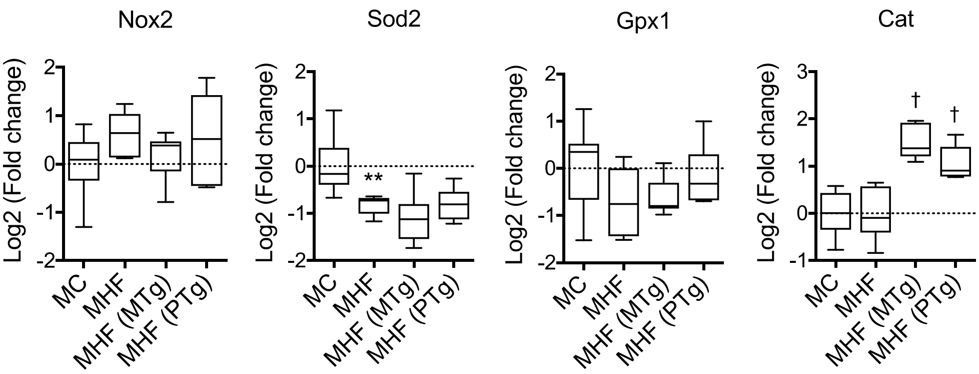

Supplement: Supplementary file 1 [file ijms-21-07342-s001.pdf]
